# G2E3 attenuating replicative stress

**DOI:** 10.18632/aging.100784

**Published:** 2015-08-18

**Authors:** Franziska Schmidt, Larry M. Karnitz, Matthias Dobbelstein

**Affiliations:** Institute of Molecular Oncology, Ernst Caspari Haus, Göttingen Center of Molecular Biosciences, GZMB Faculty of Medicine, University of Göttingen, Göttingen, Germany

Cancer cells generally display an increased level of replicative stress. While in S phase, their replication forks undergo temporary stalling and sometimes even permanent collapse, leading to the accumulation of single stranded DNA and a DNA damage response. Strategies to exploit this phenomenon by further increasing replicative stress include the use of conventional DNA-damaging chemotherapeutics, but also the targeted inhibition of damage response signaling [1].

The proteasome represents an additional target for cancer therapy. Direct inhibitors of the protease activitie(s) within the proteasome currently represent the mainstay of this approach, but chaperones of proteasome maturation may represent additional future targets.

Would it be possible that the two machineries – for DNA replication and for protein turnover – communicate with each other, with each of them relying on the other? Such interdependence has long been noticed, e. g. the degradation of CDC25A in response to DNA damage. Proteasome inhibition also depletes the cell of free ubiquitin, thus interfering with protein ubiquitination in general. As a result, signaling pathways that rely on the transfer of ubiquitin are largely inactivated. Strikingly, interfering with the ubiquitin signaling system leads to dysfunctional DNA damage response. This initial observation led to the discovery of numerous ubiquitin-dependent factors essential for DNA damage response, DNA repair, and DNA replication. Thus, besides phosphorylation, ubiquitination represents another key signaling mechanism employed by the cell to maintain the integrity of the DNA.

The Dobbelstein lab therefore performed a comprehensive siRNA screen to interrogate the functions of known ubiquitin ligases and deubiquitinating enzymes in the DNA damage response to cisplatin treatment. Platinum compounds are among the most widely used anticancer drugs. They covalently attach to the DNA to form crosslinks, some of which reach across the double helix – termed interstrand crosslinks (ICLs). Such ICLs represent major obstacles to DNA replication and require a sophisticated repair mechanism that involves retraction of the replication fork, activation the Fanconi anemia repair factors, and mobilization of homologous recombination repair [2].

The screen revealed ubiquitin ligases that, when knocked down, reduced the extent of phospho-H2AX accumulation [3]. Among the hits were siRNAs targeting proteasomal subunits, Mdm2, and Mdm4. In these cases, we suspected that the activation of p53 (by preventing its proteasomal degradation) led to cell cycle arrest, thereby decreasing the odds by that DNA replication forks hit ICLs.

However, the screen also revealed a less expected target, the ubiquitin ligase G2E3 (named after its E3 ubiquitin ligase activity and its accumulation in the G2 phase of the cell cycle [4]). When G2E3 was knocked down with multiple siRNAs, cisplatin-induced phospho-H2AX levels were consistently reduced. We therefore suspected that the removal of G2E3 attenuated the cellular response to replicative stress. And indeed, depletion of G2E3 reduced the amount of phosphorylated (and hence active) Chk1, one of the principle mediators of the response to replicative stress, in cisplatin-treated cells.

The reduction in the replicative stress response initially made us believe that removing G2E3 might facilitate the survival of tumor cells. However, the opposite was observed. Knocking down G2E3 actually decreased cell proliferation and increased caspase activity and apoptosis. Thus, G2E3 acts as a survival factor. We speculate that this pro-survival function might be triggered, at least in part, by promoting Chk1 activity.

G2E3 is essential for embryonal development [5]. It was previously implicated into the DNA damage response, mostly based on its differential cellular location upon DNA damage [4]. Along a similar line, we found that DNA damage triggers a profound reduction in G2E3 levels [3]. Thus, it seems that G2E3 is not only a regulator of the replicative stress response, but also is in itself a subject to regulation by this signaling cascade.

Open questions remain, such as the still-elusive substrate proteins that are targeted for ubiquitination by G2E3. Previous reports have only revealed a general E3 ubiquitin ligase activity for G2E3 that polymerizes ubiquitin *in vitro* as a function of its RING/PHD domains but not its HECT domain [5]. Further questions include the druggability of G2E3. At least in principle, eliminating its function might increase replicative stress and the apoptotic response in tumor cells.

**Figure 1:**
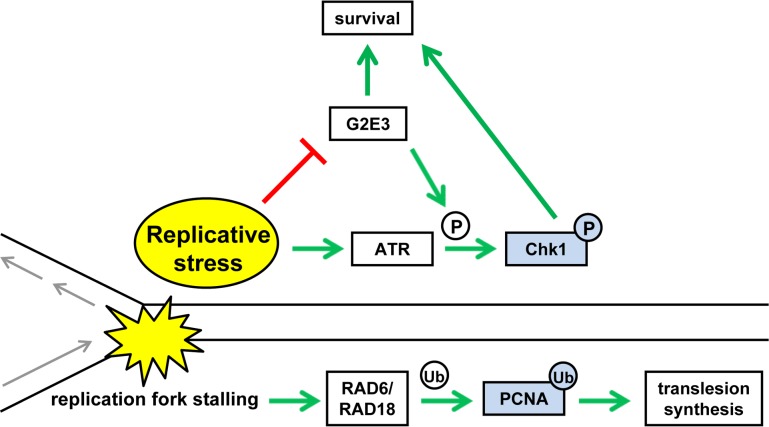
Molecular communication between G2E3 and the replicative stress response When a DNA replication fork hits a damaged site, e. g. crosslinked nucleotides, a response to replicative stress is triggered. The ubiquitination of PCNA is part of this response and enables translesion DNA synthesis. Our recently published results [3] suggest that another ubiquitin ligase, G2E3, governs the cellular response to replicative stress as well, through activation of the ATR and Chk1 kinase pathway. Both ubiquitination events thus attenuate replicative stress and support cell survival. Figure adapted and expanded from [3].

The concept of replicative stress being directly affected by ubiquitin ligases is not unheard of. For example, PCNA is ubiquitinated by the RING E3 ligase RAD18, a modification that triggers the translesion synthesis pathway, which facilitates the bypass of lesions that block DNA polymerase progression [6]. Similarly, ubiquitination regulates the activation of the Fanconi anemia (FA) pathway, which also promotes replication fork progression by coordinating the repair of ICLs that block DNA polymerase progression. ICLs that block DNA replication activate a core complex of multiple FA proteins, including the E3 ligase FANCL, which ubiquitinates the FANCD2/FANCI complex [7]. The ubiquitinated FANCD2/FANCI complex localizes to the ICL, recruits nucleases that cleave on opposites side of the lesion, and ultimately coordinates the completion of the repair by elements of the TLS and homologous recombination repair pathways. Collectively, these ubiquitinations promote the replication of the genome during periods of replication stress, and disruption of these pathways leads to enhanced replication stress. G2E3 may represent a new component of this machinery.

These studies suggest that regulation of cellular metabolism by mutp53 may be complex, and depend strongly upon the type of tissue, the “geographical” metabolic heterogeneity of tumor cells as well as the stage of tumor initiation/progression. Finally, we have reported additional key activities of SLC25A1 that are relevant to oncogenesis, consisting of regulation of mitochondrial autophagy, inflammation as well as of the intracellular pools of Acetyl-Coenzyme A, the donor for all trans-acetylation reactions [5,8]. Therefore, SLC25A1 likely provides a nodal point at the cross-road of multiple signal pathways altered by mutant p53.
